# G0 or no-G0: phosphatase control of quiescence and cell cycle entry

**DOI:** 10.1042/BST20250091

**Published:** 2026-05-21

**Authors:** Eleanor A. Robinson, Alexis R. Barr

**Affiliations:** 1 Institute of Clinical Sciences, Imperial College London, London W12 0HS, U.K.; 2 MRC Laboratory of Medical Sciences (MRC LMS), Du Cane Road, London W12 0HS, U.K.

**Keywords:** cell cycle, cell proliferation, protein phosphatases, quiescence, signalling

## Abstract

During each cell cycle, cells must decide whether to continue to proliferate or to exit the cell cycle into a reversible arrest state, known as quiescence, or G0. This decision must be highly regulated to ensure proper tissue homeostasis. Studies on kinase-driven signalling pathways that regulate this decision point have dominated the field, and the role of phosphatases remains comparatively underexplored, yet the role of phosphatases is vitally important in signal transduction. In the present review, we examine how phosphatases contribute to the regulation of quiescence in mammalian cells across three stages: entry into quiescence, maintenance of the quiescent state, and quiescence exit into the cell cycle. We discuss how phosphatases counteract mitogenic signalling pathways, including MAPK/ERK and PI3K-AKT-mTOR, and maintain low cyclin-dependent kinase (CDK) activity through dephosphorylation of key cell cycle regulators, such as the retinoblastoma family proteins and CDK inhibitors. Finally, we highlight emerging evidence that dynamic regulation of phosphatase activity shapes the transition from quiescence back into proliferation. Understanding how phosphatases regulate the reversible nature of cell cycle arrest is important for understanding how tissues maintain homeostasis and how dysregulation of quiescence contributes to disease, including cancer.

## Introduction

Quiescence (or G0) is often referred to as the ‘resting’ phase of the mammalian cell cycle and is a reversible state of cell cycle arrest. Quiescent cells remain responsive to external conditions and are primed to re-enter the cell cycle. Although quiescence encompasses diverse cellular states [1,2], quiescent cells are typically diploid and have low activity of the cyclin-dependent kinases (CDKs), the enzymes that drive entry into and progression through the cell cycle [3,4].

Most cells in the body reside in a quiescent state, and precise regulation of entry and exit is essential to maintain tissue homeostasis and prevent uncontrolled proliferation. Adult stem cells are frequently maintained in quiescence [5–7], which minimises accumulation of DNA damage from replication stress and slows cellular ageing [8]. If quiescence is improperly regulated, stem cell pools can become prematurely exhausted [9], compromising tissue maintenance and repair. Experimentally, quiescence can be induced by contact inhibition, mitogen withdrawal, serum starvation, or pharmacological inhibition [1,4,10]. Cells also enter quiescence spontaneously in response to intrinsic signals, such as replication stress [11–14]. The microenvironment further shapes quiescence through signals from the niche, cell adhesion, and hypoxia [1]. Even in hyper-proliferative contexts, such as cancer, a fraction of cells will occupy a non-proliferative quiescent state [15–17]. These cells can evade conventional chemotherapeutics that target cycling cells, enabling their survival and potential for relapse [18–20]. Understanding the mechanisms that regulate quiescence informs strategies to target these resistant cells.

**Figure 1:**
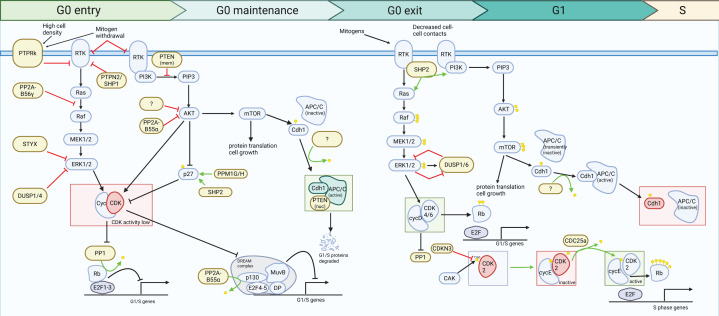
Overview of phosphatases controlling G0 entry, maintenance, and exit towards G1/S Schematic overview of signalling pathways regulating transitions between quiescence (G0) and cell cycle re-entry. The diagram is organised into four phases: G0 entry, G0 maintenance, G0 exit, and progression through G1 towards S phase (cell cycle re-entry). During G0 entry, mitogen withdrawal and high cell density reduce receptor tyrosine kinase (RTK) signalling. Negative regulators such as PP2A, PTPs, DUSP1/4, and STYX inhibit Ras-MAPK and AKT signalling pathways, resulting in reduced CDK activity. Rb is dephosphorylated by PP1, repressing E2F-dependent transcription and suppressing G1/S gene expression. During G0 maintenance, transcriptional repression deepens with the formation of the DREAM (dimerization partner (DP), p130, E2F, and multi-vulval class B (muvB)) complex. Protein synthesis is reduced through decreased mTOR activity and the activity of an ubiquitin ligase, APC/C^Cdh1^, which ensures continuous degradation of key cell cycle regulators, re-enforcing the quiescent state. PTEN has two key functions, enzymatically opposing and inhibiting PI3K proliferative signalling and a scaffolding role to promote APC/C^Cdh1^ complex formation. CDK activity is further repressed through the stabilisation of the CDK inhibitor p27, promoted by the removal of destabilising phosphorylation by SHP2, PPM1G, and PPM1H. Upon G0 exit, mitogen stimulation and decreased cell-cell contact reactivate RTK signalling, leading to activation of Ras-RAF-MEK-ERK and PI3K pathways. DUSP1 and DUSP6 are up-regulated on mitogenic stimulation and act to restrain ERK signalling in a negative feedback loop. SHP2 promotes the activation downstream of RTK signalling. This results in cyclin expression, CDK activity, inhibition of PP1, phosphorylation of Rb, and release of E2F transcription factors. The first step in CDK activation is the phosphorylation of the T-loop by CDK-activating kinase (CAK); for CDK2, this is opposed by the phosphatase CDKN3. As cells progress through G1, accumulation of Cyclin–CDK complexes drive furthers Rb phosphorylation and inactivation of APC/C^Cdh1^. This promotes the transcription of a CDK-activating phosphatase, CDC25A, promoting higher CDK activity and allowing cells to commit to DNA replication (S phase). Green arrows indicate activation, while blunt-ended red lines indicate inhibitory interactions. Phosphate groups are represented by yellow circles. Phosphatases discussed in the present review are highlighted in yellow, and unidentified phosphatases are represented with a question mark. Figure created with BioRender.com

Cell cycle entry from quiescence is driven by progressive activation of CDKs. CDK activity requires association with cyclins, whose expression oscillates. In early G1, D-type cyclins are expressed following mitogenic stimulation and bind CDK4 and CDK6. Cyclin D–CDK4/6 complexes phosphorylate retinoblastoma (Rb) family proteins, which include Rb, p130, and p107 [21–24]. Phosphorylation of these proteins relieves repression of E2F transcription factors, enabling E2F-dependent gene expression [24]. The resulting transcriptional programme promotes further cell cycle progression, including the induction of Cyclin E and the CDK-activating phosphatase, CDC25A. This increases CDK2 activity, which drives Rb hyperphosphorylation and establishes a positive feedback loop to reinforce E2F activity [24,25]. Rising CDK2 activity also promotes inactivation of the anaphase-promoting complex/cyclosome (APC/C) [26,27], a key E3 ubiquitin ligase that targets numerous cell cycle regulators for degradation, including Cyclin A, CDC25A, and Skp2 [28–30]. In quiescence and G1, the APC/C is active in association with its co-activator Cdh1. Inactivation of APC/C^Cdh1^ allows stabilisation of its targets, further amplifying CDK activity and facilitating progression into S phase, where DNA replication occurs. The transition from quiescence to proliferation therefore depends on the accumulation of phosphorylated CDK substrates. Conversely, quiescence requires the establishment and maintenance of a hypophosphorylated cell cycle network, sustained not only by low CDK activity but also through active phosphatase-mediated dephosphorylation. During exit from quiescence, phosphatases can assume an opposing role, supporting kinase signalling to promote CDK activation and cell-cycle re-entry. This highlights that phosphatases contribute to both restraining and facilitating proliferation, depending on cell cycle stage (Figure 1).

Phosphorylation is one of the most common post-translational modifications used to modulate protein behaviour, occurring on over two-thirds of human proteins [31]. Once added, phosphate groups are extremely stable [32], so the reversibility of this modification depends on the action of phosphatases. Dephosphorylation allows for rapid reversal of kinase-driven signalling instead of the slower protein degradation and resynthesis. The net phosphorylation state of a substrate depends on the balance between kinase and phosphatase activities, allowing for the integration of multiple signalling inputs. During the cell cycle, many phosphorylation events must occur in a precise order, and phosphatase activity is important for regulating the timing of substrate phosphorylation [33,34]. Advances such as live-cell imaging, computational substrate prediction [35], targeted inhibition [36], and new methods for substrate-specific dephosphorylation [37,38] now allow deeper exploration of these mechanisms.

The human phosphatome is encoded by an estimated 189 genes, compared with 538 kinases [39]. Phosphatases achieve substrate specificity largely through regulatory subunits, which guide the activity of catalytic subunits. They are structurally and functionally diverse, spanning around 20 distinct families [39] including protein phosphatases, non-protein phosphatases, and even pseudophosphatases, which often have scaffolding roles.

While thousands of phosphorylation sites are linked to kinases, only ∼7% are linked to a specific phosphatase [39]. Understanding which phosphatases act at defined signalling nodes is essential for a proper understanding of cell cycle regulation and is highly relevant therapeutically, as kinase inhibition will be shaped by opposing phosphatase activity. Considerable research has mapped kinase-driven cell cycle entry, but the phosphatases that counteract these signals remain comparatively under-characterised.

In the present review, we focus on the role of phosphatases in the life cycle of quiescence. We begin by discussing how cells enter G0, then discuss how the arrested state is maintained, and finally examine the processes controlling exit from quiescence and re-entry into the cell cycle.

## Entry into G0

### Mitogenic withdrawal and Rb dephosphorylation

Entry into quiescence is initiated when CDK activity declines and phosphatase activity predominates. Mitogenic signalling normally sustains CDK activity through MAPK-ERK-dependent induction of cyclin transcription [40]. When these inputs fall, the balance shifts towards dephosphorylation of CDK substrates (Figure 1). Some of the most important substrates here are the pocket proteins: Rb, p107, and p130. In quiescent cells, phosphatases maintain the pocket proteins in a hypophosphorylated, transcriptionally repressive state, preventing proliferation. Cells lacking all pocket proteins fail to respond appropriately to quiescence-inducing cues [41,42].

Despite structural similarity, the three pocket proteins play distinct roles in quiescence. p107 is not considered further here, as it is minimally expressed in quiescent cells and shows little to no promoter occupancy during quiescence [43,44]. Rb is the most extensively characterised pocket protein, and when hypophosphorylated, Rb binds to E2F transcription factors 1-3 [45,46], preventing transcription of genes necessary for cell cycle progression [47]. Protein phosphatase 1 (PP1) was identified as the main phosphatase responsible for Rb dephosphorylation [48–50]. Uniquely, PP1 binds directly to Rb through its catalytic subunit [51] via an RVxF-like docking motif. This binding site overlaps with the CDK docking site [52] and is independent of Rb phosphorylation status [51], creating a competitive binding site. Rb dephosphorylation begins at anaphase, during mitotic exit [48,53], when falling CDK1 activity permits autocatalytic removal of the inhibitory T320 phosphorylation on PP1, resulting in increased PP1 activity [54–56]. Initially it was thought that Rb was fully dephosphorylated upon mitotic exit, yet more recently it has been shown that only a partial reset occurs at this stage [16]. Complete dephosphorylation of Rb occurs only when CDK activity remains persistently low, such as during quiescence where low CDK states are re-enforced.

### Contact inhibition and suppression of RTK signalling

Quiescence can also be induced in the continued presence of growth factors, most notably during contact inhibition. In this context, cells must actively attenuate mitogen-driven signalling despite extracellular ligand availability. Cell density-dependent signalling ultimately converges on the same downstream pathways as mitogen withdrawal (Figure 1), with suppression of CDK activity and the phosphorylation state of Rb. However, transcriptional profiling reveals that different quiescence-inducing stimuli produce distinct quiescence states [4]. Mitogens activate RTKs, which stimulate downstream MAPK-ERK signalling to promote transcription of cell cycle genes, including cyclins (Figure 1). Protein tyrosine phosphatases (PTPs) play a key role in restraining RTK activity under conditions of high cell density by dephosphorylating and inactivating RTKs [57,58]. The influence of cell density extends to daughter cell fate, such that prior high-density exposure predisposes cells towards lower CDK activity in the subsequent generation [59].

Some PTPs span the cell membrane and are classified as receptor-like PTPs (R-PTPs), many of which are up-regulated in response to increased cell-cell contacts [60–62]. PTPR-kappa (PTPRk) is one such example, and its expression increases at high cell density [63–65]. PTPRk up-regulation forms a core part of the quiescence programme and acts as a convergence point across different quiescence-inducing stimuli [4]. PTPRk is required for contact-inhibited growth arrest and was initially proposed to dephosphorylate epidermal growth factor receptor (EGFR) [65]. However, its effects on EGFR signalling appear independent of its catalytic activity [66], suggesting important scaffolding roles instead. PTPRk activity reduces CDK activity during quiescence by regulating the CDK inhibitors p27(Kip1) and p21(Cip1/Waf1). Depleting PTPRk reduces both these CDK inhibitors, while overexpression results in decreased CDK2 activity [65]. However, the contribution of the phosphatase activity compared with the scaffolding role has not yet been deconvoluted. Cytosolic PTPs have been described to modulate RTK signalling, for example, PTPN2 which dephosphorylates EGFR to limit downstream protein kinase B (AKT) activity [67]. RTK signalling can be restrained further downstream by the activity of Src homology region 2 domain-containing phosphatase-1 (SHP-1), which dephosphorylates and inactivates JAK2 and STAT3, leading to down-regulation of proliferation-associated targets, including Cyclin D [68,69].

Members of the dual-specificity phosphatase (DUSP) family can dephosphorylate both tyrosine and serine/threonine residues and include the MAPK phosphatases (MKPs), which regulate ERK, JNK, and p38 signalling [70]. MKPs can be classified based on localisation and substrate specificity [71]. In confluent cells, DUSP1 and DUSP4 have been shown to limit ERK1/2 activity [72]. Consistently, extracts from confluent cells inactivate ERK1/2 more efficiently than those from sparse cultures, and treatment with the PTP inhibitor sodium orthovanadate can reverse this inhibition [72], although some of this effect may be due to upstream R-PTPs. Overexpression of DUSP1 can prevent quiescent fibroblasts from re-entering S phase, even in the presence of growth factors [73], highlighting the role of ERK dephosphorylation in quiescence entry. The DUSP family also contains pseudophosphatases, such as STYX, which lack catalytic activity but can modulate signalling by acting as scaffolds, anchors, or competitive inhibitors [74]. STYX acts as a nuclear anchor for ERK1/2, limiting cytosolic reactivation by MEK1/2, therefore reducing ERK1/2 signalling (Figure 1) [75]. The role of STYX in regulating other MAPKs is unknown [74].

When cells enter quiescence at high cell density, p27 accumulates in the nucleus, where it inhibits CDK activity [59]. Both the localisation and stability of p27 are regulated by phosphorylation [76]. CDK-mediated phosphorylation of T187 in p27 promotes recognition of p27 by Skp2, a ubiquitin ligase, and subsequent degradation [76,77]. The phosphorylation of T198 in p27 by AKT is thought to promote p27 nuclear export [78]. Despite the central role of p27 in quiescence stabilisation, the importance of phosphatases for p27 regulation is not yet fully established. Two members of the protein phosphatase metal-dependent (PPM) family have been proposed to oppose CDK- and AKT-dependent p27 phosphorylation. PPM1H is suggested to dephosphorylate pT187, stabilising p27 against degradation, while PPM1G is thought to act on pT198, promoting nuclear retention [79,80]. The effects of altering PPM1H levels or activity on the cell cycle have not yet been explored, so it remains unclear whether this phosphatase is required for quiescence maintenance. PPM1G depletion has been shown to reduce G1 arrest in response to contact inhibition in HeLa cells, but its role in quiescence in non-transformed cells has not been established. Similar to PPM1H, the PP2A-B56γ holoenzyme has been reported to target pT187 [81]. PP2A-B56γ has also been proposed to dephosphorylate pT157 in the nuclear localisation signal (NLS), further promoting nuclear accumulation [81,82]. In addition to serine/threonine modifications, p27 can be destabilised by tyrosine phosphorylation downstream of active RTK signalling [83–86]. However, the phosphatases responsible for reversing these modifications remain largely undefined. To date, only one phosphatase, SHP2, has been linked to p27 tyrosine dephosphorylation. Overexpressing SHP2 in cells delays p27 degradation, yet the molecular mechanism underlying this stabilisation remains unclear [87].

### Endogenous replication stress

Quiescence entry can be driven by endogenous DNA damage arising from replication stress during the previous S phase [11–14]. The role of phosphatases in the DNA damage response has been reviewed extensively elsewhere [88,89]. Here we focus on phosphatases in quiescence entry downstream of endogenous DNA damage. Endogenous DNA damage triggers p53-dependent accumulation of p21, which is inherited by daughter cells, where it inhibits CDK activity, resulting in quiescence entry. For quiescence entry to occur, DNA damage signalling must be of sufficient amplitude to overcome the opposing activity of phosphatases that act to terminate the response.

WIP1 (*PPM1D*), a transcriptional target of p53 [90], acts as a brake on the DNA damage response (DDR) pathway. WIP1 engages in a negative feedback loop with p53, destabilising p53 through direct dephosphorylation and by promoting MDM2-mediated degradation [91,92]. Through this, WIP1 indirectly suppresses p21 levels to oppose quiescence entry. Protein phosphatase 4 (PP4) also plays a role in the DDR. PP4 inhibits the transcription of p21 (*CDKN1A*) through the dephosphorylation of KAP1, a transcriptional repressor, to oppose the DDR effector kinases ATM and CHK2 [93]. Damage signalling must be robust enough to overcome PP4 activity, to sustain inhibitory KAP1 phosphorylation, and to facilitate p21 transcription for quiescence entry.

## Maintenance of G0

### Transcriptional repression enforced by phosphatases

Maintenance of quiescence requires sustained repression of cell cycle genes, a role primarily fulfilled by the pocket protein, p130. While Rb levels remain largely constant, p130 is specifically up-regulated during quiescence. Dephosphorylated p130 most often binds to E2F4, which unlike E2F1–3 functions as a transcriptional repressor. The p130–E2F4 complex occupies promoters during quiescence, preventing cell cycle gene transcription [44,94–96]. E2F4 lacks an intrinsic NLS, so p130 plays a critical role in the nuclear retention of the complex [97]. These p130–E2F4 complexes recruit chromatin-modifying co-repressors [98], including components of the DREAM complex, to enforce long-term silencing of cell cycle genes, such as E2F1–3 and CDC25A, during quiescence. In addition to its transcriptional role, p130 possesses CDK inhibitory activity through its N-terminal region, further reinforcing cell cycle exit.

The phosphorylation state of p130 is a key determinant of its function and stability. Unlike Rb, p130 is not a major substrate of PP1 [99,100]. Instead, its phosphorylation state is thought to be primarily regulated by protein phosphatase 2A (PP2A). PP2A is a serine/threonine phosphatase that forms a trimeric complex comprising a catalytic subunit (PP2A/C), a scaffolding subunit (PP2A/A), and regulatory subunits (PP2A/B) that confer substrate specificity [101]. CDK4–Cyclin D complexes phosphorylate p130, preventing its association with E2F4 and relieving transcriptional repression [102], and PP2A is proposed to counteract these phosphorylations. However, overexpression of PP2A-B55α in cycling cells results in only partial dephosphorylation of p130 [103], suggesting that additional phosphatases or alternative PP2A regulatory subunits may contribute to its regulation. The specificity of different PP2A/B subunits for p130 remains unexplored; so far only the holoenzyme PP2A-B55α has been shown to interact with p130, including in quiescent cells [99,103]. While it is assumed that PP2A can counteract the 22 identified CDK phosphorylation sites on p130 [102], these sites have not been systematically tested for dephosphorylation, and most evidence comes from studies with purified proteins rather than *in vivo* analyses. Consequently, it remains unclear whether PP2A alone is sufficient to fully oppose CDK-mediated phosphorylation of p130 or how this influences DREAM complex stability in G0. Phosphorylation also regulates p130 stability, as phosphorylated p130 is targeted for degradation, while PP2A may promote nuclear retention by dephosphorylating sites that expose the NLS [104,105]. Taken together, PP2A-mediated dephosphorylation of CDK sites maintains p130 in a transcriptionally repressive state.

Beyond its activity during quiescence, PP2A also appears to regulate quiescence at an earlier stage, involving a different regulatory subunit, B56γ. The activity of the PP2A-B56γ holoenzyme is required during G2 to enable stable entry into quiescence [106]. Inhibition of PP2A-B56γ in G2 reduces DREAM complex formation and limits its recruitment to chromatin in subsequent quiescence. Loss of PP2A-B56γ is thought to hyperactivate Ras signalling during G2, leading to premature up-regulation of Cyclin E and a CDK2-high state in mitosis, which predisposes cells to escape quiescence. The specific substrates of PP2A-B56γ in Ras signalling remain unidentified [106].

### PTEN and suppression of PI3K-AKT in G0

The phosphatase and tensin homolog (PTEN) is a key tumour suppressor gene with essential roles in restraining cell growth [107]. PTEN shares catalytic homology with the PTP family [39]; however, unlike most members of this family, PTEN acts as both a lipid and protein phosphatase [108–110]. At the cell membrane, PTEN catalyses the hydrolysis of phosphatidylinositol-3,4,5-triphosphate (PIP3) to phosphatidylinositol 4,5-bisphosphate (PIP2). In this way, PTEN opposes phosphoinositide 3-kinase (PI3K), which generates PIP3 in response to mitogenic signalling. PIP3 acts as a pro-proliferative lipid messenger that promotes activation of AKT and downstream pathways controlling growth and survival. Loss of PTEN function is frequently observed in cancers [111], resulting in hyperactivation of the PI3K-AKT pathway and a failure to maintain quiescence [112]. When AKT signalling is dampened by PTEN, p27 can accumulate in the nucleus to maintain the CDK-low state [113,114].

The activity of AKT is directly regulated by its phosphorylation status at two key sites: T308, located in the activation loop and phosphorylated by 3-phosphoinositide-dependent kinase-1, and S473, located in the hydrophobic motif and phosphorylated by the mechanistic target of rapamycin complex 2 [115,116]. Consequently, the dephosphorylation of these sites acts to negatively regulate AKT activity (Figure 1), though the identities of the responsible phosphatases have been subject to debate. PP2A has been implicated in the dephosphorylation of T308, with knockdown of the regulatory subunit B55α [117] resulting in elevated phosphorylation at this site. Dephosphorylation of S473 was originally attributed to the PH domain leucine-rich repeat-containing protein phosphatase family (PHLPP1/2) [118,119]. However, PHLPP2 has since been shown to lack catalytic activity, and ancestral sequence analysis suggests that PHLPP1 is likely also a pseudophosphatase [120]. As a result, the identity of the phosphatase responsible for S473 dephosphorylation remains unresolved. PP2A-B56β has been proposed to fill this role [120], acting on both S473 and T308, while B55α shows limited activity towards S473.

Phosphorylation, particularly at the C-terminal tail, has been shown to influence the activity and stability of PTEN [121–123]. The kinases casein kinase 2 and glycogen synthase kinase 3β have been shown to phosphorylate this region [124–126]. Dephosphorylation of these sites is associated with increased PTEN activity, a crucial function within cells, yet the phosphatases responsible remain unidentified. Beyond its membrane-associated role, PTEN exhibits nuclear localisation during quiescence [127]. Here, PTEN can promote cell cycle arrest independently of AKT inhibition [128,129] and its catalytic activity. Nuclear PTEN has been shown to facilitate association of the APC/C with Cdh1 [130], reinforcing a CDK-low state, albeit in a phosphatase activity-independent manner. Consistent with this, expression of phosphatase-inactive PTEN delays S phase entry.

## Exit from G0

Upon receiving appropriate proliferative signals, cells exit quiescence back into the cell cycle. Cells do not ‘reawaken’ from quiescence uniformly, reflecting the collection of different states and depths within the umbrella term quiescence [4]. Some cells can be poised to re-enter proliferation rapidly, whereas others require more extensive reactivation [131]. The first G1 following quiescence exit often progresses more slowly than in continuously cycling cells [132], reflecting the need to re-establish transcriptional, translational, and metabolic programmes. Thus, exit from quiescence is not simply a reversal of the entry process but a highly ordered transition in which signalling shifts from a phosphatase-dominated state towards increased kinase activity. Not all cells exit successfully; some are directed towards senescence or cell death.

Although thus far in the present review phosphatases have been discussed as inhibitors of proliferation, they also actively contribute to re-entry back into the cell cycle. For example, CDC25A and SHP2 enhance proliferative signalling by activating CDKs or reinforcing growth factor pathways [133,134]. Given the central role of phosphorylation in regulating quiescence exit and the need for dynamic signalling networks to respond dynamically to intrinsic and extrinsic cellular cues, it is therefore likely that additional phosphatases participate in this process, coordinating the timing and order of events to ensure that cells only commit to proliferation under appropriate conditions. However, relatively few phosphatases have been characterised in this context, leaving room for further exploration of phosphatases in quiescence exit.

### Regulation of APC/C^Cdh1^ during cell cycle re-entry

In quiescence, APC/C is bound to its co-activator Cdh1, forming APC/C^Cdh1^, which maintains a CDK-low state and degrades substrates such as Cyclin A, CDC25A, and Skp2. This activity is essential for maintaining quiescence, and Cdh1 must be kept dephosphorylated to allow APC/C^Cdh1^ function [135]. In budding yeast, a key mitotic exit phosphatase, Cdc14, activates Cdh1, but a comparable mechanism for maintaining Cdh1 activity in quiescence in mammalian cells has not yet been identified [136,137].

Control over APC/C^Cdh1^ activity on exit from quiescence has been shown to couple the metabolic state of a cell to cell cycle progression [138]. Recently, it has been shown that APC/C^Cdh1^ is temporarily inactivated during exit from quiescence by mTOR-dependent Cdh1 phosphorylation. The transient decrease of APC/C^Cdh1^ activity allows for the accumulation of its substrates, including metabolic enzymes, to promote an increase in available ATP, providing energy for cell growth in G1 [138]. APC/C^Cdh1^ must then be reactivated and remain active until the G1/S transition, ensuring correct progression into S phase. While it was shown that phosphatase activity is necessary for APC/C^Cdh1^ reactivation, the phosphatase responsible has yet to be identified.

Regulation of APC/C^Cdh1^ activity is critical throughout quiescence and G1, to such an extent that this ubiquitin ligase may act as the ultimate gatekeeper of quiescence. Cells with phosphorylated Rb are still able to enter mitogen-sensitive quiescence, yet when the APC/C^Cdh1^ is inactivated, this reversibility is lost, and cells are committed to progress into DNA replication [27]. The stringency of this control implies that APC/C^Cdh1^ activity must be tightly regulated, not only through CDK-dependent phosphorylation but likely also through phosphatase-mediated mechanisms. Such regulation would ensure both the maintenance of quiescence and the precise timing of irreversible cell cycle commitment.

### Mitogenic signalling and the kinase-phosphatase switch

Cells that entered quiescence due to loss of mitogenic signalling will reactivate RTK pathways. In the context of contact inhibition, reduced cell density relieves inhibitory cell-cell interactions such as those established by R-PTPs. Together, ERK/MAPK and PI3K-AKT-mTOR pathways are restored, resulting in restored transcriptional and translational capacity and the accumulation of cyclins required for CDK activation.

One of the most studied PTPs is SHP2 (encoded by *PTPN11*), which was the first identified proto-oncogenic PTP and a prominent target in cancer research [139,140]. SHP2 acts downstream of most RTKs [141], where its SH2 domains bind phosphotyrosine motifs on activated receptors or adaptor proteins, relieving its autoinhibition and activating its catalytic domain. SHP2 functions broadly as a positive regulator of mitogenic signalling by promoting activation of key proliferative pathways, most notably Ras/ERK1/2 and PI3K/AKT (Figure 1). SHP2 has been shown to dephosphorylate and directly activate Ras, while negatively regulating Ras inhibitors such as Sprouty [142]. Large-scale phosphoproteomics have identified multiple new SHP2 targets contributing to its downstream positive regulation of EGFR signalling [143]. Functionally, loss of SHP2 delays cell cycle progression, with SHP2 depletion extending G1 phase in cancer cell lines [144]. SHP2 gain-of-function mutations elevate MAPK and PI3K signalling and promote proliferation and survival in multiple cancer contexts [134].

Mitogenic stimulation also induces transcription of several members of the DUSP family, creating a negative feedback loop that shapes the amplitude and duration of ERK signalling at quiescence exit. This is important because while ERK activity must reach a sufficient level to drive cell cycle entry, sustained overactivation can direct cells towards apoptosis in some cell types [145,146]. DUSP1, encoded by an immediate-early gene, is rapidly transcribed following mitogenic stimulation and restrains nuclear ERK activity [72,147,148]. ERK reciprocally promotes ubiquitin-mediated DUSP1 degradation, establishing a double-negative feedback loop, regulating the extent of its own supression [149]. DUSP6 is similarly induced and activated downstream of ERK activity [150,151], also acting to dephosphorylate and inactivate ERK activity. The DUSPs therefore represent actively regulated phosphatases at the quiescence exit transition (Figure 1), ensuring ERK activity is of sufficient amplitude and duration to commit cells to G1 entry.

The downstream consequence of restored mitogenic signalling is the activation of CDKs, directly driving cell cycle re-entry. In addition to cycling binding, full CDK activation requires T-loop phosphorylation of each CDK (CDK1 at T161, CDK2 at T160, CDK4 at T172, and CDK6 at T177 [152]). T-loop phosphorylation is catalysed by the CAK, a complex of CDK7, Cyclin H, and Mat1 [153]. The accessibility of the T-loop after phosphorylation differs across CDK complexes; CDK4/6 T-loop phosphorylation is rapidly reversed upon CDK7 inactivation, whereas CDK1/2 T-loop phosphorylation persists with extended CDK7 inactivation [154]. The phosphorylated residue in the T-loop of CDK1/2 is protected from phosphatase activity when in the cyclin-bound active conformation [154,155]. CDK4/6 activity is therefore particularly sensitive to the balance between CAK and opposing phosphatase activity, and mitogenic stimulation promotes re-entry by shifting this balance in favour of phosphorylation. This is consistent with the observed increase in CDK4 T172 phosphorylation upon growth factor stimulation [156]. The phosphatase that dephosphorylates the T-loop of CDK2, when unbound from Cyclin A, is cyclin-dependent kinase inhibitor 3 (CDKN3, also known as KAP) [155,157–159]. CDKN3 does not interact with CDK4/6 [158,159], leaving the identity of the phosphatase responsible for the rapid dephosphorylation of the CDK4/6 T-loop unresolved.

### Transcriptional reactivation

In quiescent cells, transcription is globally repressed through DREAM complexes and down-regulation of RNA, DNA, and protein biosynthesis [1]. Yet cells can still rapidly reactivate transcription of key genes to switch back into the proliferative cycle. Promoter-proximal pausing of RNA polymerase II (Pol II) at key genes, such as those involved in RNA biosynthesis, keeps them transcriptionally ‘primed’ for rapid reactivation [160] during quiescence exit. Release of paused Pol II into productive mRNA elongation is a key regulatory step during cell cycle re-entry. This process is controlled by the kinase activity of the positive transcription elongation factor b (P-TEFb), a complex of CDK9 and Cyclin T1 [161,162]. P-TEFb phosphorylates key negative regulators of elongation, including SPT5, a component of the DRB sensitivity-inducing factor, as well as the negative elongation factor (NELF). Phosphorylation of SPT5 at S666 reduces its inhibitory function, while the phosphorylation of NELF promotes its dissociation from paused Pol II, together enabling elongation to proceed [163–165]. This release step is tightly opposed by members of the phosphoprotein phosphatase family, ensuring strict pause release timing. PP4 directly counteracts P-TEFb activity by dephosphorylating SPT5 at S666, thereby reinforcing promoter-proximal pausing [166]. Depletion of the PP4 regulatory subunit PP4R2 leads to the loss of promoter-proximal pausing. PP2A has also been implicated in regulating pause release through the same dephosphorylation site [167]. Depletion of PP2A catalytic or scaffold subunits enhances Pol II pause-release. Proteomics analysis identified INTS8, a component of the Integrator complex, acting here as a substitute PP2A regulatory subunit. Knocking down INTS8 mimics the results observed with PP2A loss [167]. The PP2A–Integrator complex also appears to dephosphorylate SPT5 at S666 [167,168], and pharmacological activation of PP2A enhances promoter-proximal pausing. Whether PP4 and PP2A act independently or cooperate to establish a phosphorylation threshold to gate P-TEFb-dependent pause release remains to be resolved.

A notable example of a paused gene in quiescence is C-terminal domain phosphatase subunit 1 (*CTDP1*), which recycles Pol II by dephosphorylating its C-terminal domain (CTD). In quiescence, *CTDP1* transcription is stalled, which restrains Pol II activity, but upon mitogenic stimulation, it can be rapidly up-regulated, facilitating further bursts of transcription. This poised state allows cells to maintain quiescence while remaining primed for efficient re-entry. Depletion of CTDP1 causes quiescence entry associated with elevated p27 and p130 expression [160], suggesting its expression is essential for G1 progression. Similarly, the fission yeast ortholog Fcp1 was identified as a quiescence regulator from a genetic screen [169]. There is evidence that PP1 may also dephosphorylate the Pol II CTD in complex with PNUTS. In human cell extracts, inactive PP1-PNUTS co-purifies with Pol II with a phosphorylated CTD [170], and *in vitro* assays demonstrate that PP1-PNUTS can dephosphorylate the Pol II CTD [171]. However, whether PP1-PNUTS directly targets the Pol II CTD *in vivo* remains unresolved.

Cell cycle gene transcription is triggered by rising CDK activity, which hyperphosphorylates Rb and p130, relieving repression of E2F-dependent genes [24,25]. One such E2F transcriptional target, CDC25A, a dual-specificity phosphatase, is critical for activating CDK2. CDC25A removes inhibitory phosphorylations at T14 and Y15 from the active site of CDK2 to activate Cyclin E/A–CDK2 complexes and drives the G1-to-S phase transition [133]. Loss of CDC25A activity results in cells arresting in G1, highlighting its essential role in cell cycle re-entry [172].

## Conclusions and future directions

Despite extensive characterisation of kinase-driven mechanisms controlling quiescence entry and exit, the phosphatase landscape governing these transitions remains remarkably underdefined. In many cases, phosphatase activity is assumed to be a passive consequence of low kinase activity, rather than an actively regulated process. Future work must move beyond kinase-centric models to define substrate-specific phosphatase networks and determine how phosphatase activity is controlled in quiescence entry, maintenance, and exit. The tools to address this are now emerging and have already proven powerful in driving understanding at other cell cycle transitions. Quantitative integration of kinase profiling and endogenous phosphatase profiling has revealed how specific kinases regulate phosphatase holoenzyme assemblies during mitosis [173]. Building on this, chemical-genetic systems now enable rapid, holoenzyme-specific inhibition of phosphatases by blocking their substrate-binding interface, the direct effects of which can be quantified with phosphoproteomics [36]. Combined with an expanding toolkit including computational substrate prediction [35], these methods make establishing a systematic map of phosphatase activity across the quiescence transition an achievable goal. This would strengthen our understanding of how cells coordinate exit and re-entry into the cell cycle.

## Perspectives

- Importance of the field: Tight control of phosphorylation governs quiescence and cell cycle re-entry, processes often dysregulated in cancer. Understanding phosphatase activity at key signalling nodes could reveal new therapeutic targets and improve current strategies.
- Current thinking: Research has largely focused on kinases, yet phosphatases act widely throughout these pathways. Many studies identify individual phosphatases, but gaps remain in substrate specificity, dephosphorylation sites, and their functional impact on cell cycle progression.
- Future directions: Systematic mapping of phosphatase-substrate relationships and integrating phosphatases into signalling network studies will clarify how site-specific phosphorylation is coordinated and may reveal the mechanisms that ensure the ordered sequence of phosphorylation events.

## Open Access

Open access for this article was enabled by the participation of Imperial College London in an all-inclusive Read & Publish agreement with Portland Press and the Biochemical Society under an agreement with JISC.

## Abbreviations

APC/C: anaphase-promoting complex/cyclosome
CDK: Cyclin-dependent kinase
CDKN3: Cyclin dependent kinase inhibitor 3
CTD: C-terminal domain
CTDP1: C-terminal domain phosphatase subunit 1
DDR: DNA damage response
DP: dimerization partner
DREAM: dimerization partner, p130, E2F, and multi-vulval class B (muvB)
DUSP: dual-specificity phosphatase
EGFR: epidermal growth factor receptor
G0: quiescence
KAP1: KRAB-associated protein 1
MAPK: mitogen-activated protein kinase
MKPs: MAPK phosphatases
NELF: negative elongation factor
NLS: nuclear localisation signal
PIP3: phosphatidylinositol-3,4,5-triphosphate
PIP2: phosphatidylinositol 4,5-bisphosphate
PTEN: phosphatase and tensin homolog
PI3K: phosphoinositide 3-kinase
P-TEFb: positive transcription elongation factor b
PP1: protein phosphatase 1
PP2A: protein phosphatase 2A
PP4: protein phosphatase 4
PPM: protein phosphatase metal-dependent
PTPs: protein tyrosine phosphatases
PTPR: receptor-like protein tyrosine phosphatase
PTPRk: PTPR-kappa
Rb: retinoblastoma
R-PTPs: receptor-like PTPs
RTK: receptor tyrosine kinase
SHP1: Src homology region 2 domain-containing phosphatase-1
SHP2: Src homology 2 domain-containing phosphatase-2
